# Current Trends in Chemotherapy in the Treatment of Metastatic Prostate Cancer

**DOI:** 10.3390/cancers15153969

**Published:** 2023-08-04

**Authors:** Janice Zhao, Brendan J. Guercio, Deepak Sahasrabudhe

**Affiliations:** James P. Wilmot Cancer Institute, University of Rochester, Rochester, NY 14642, USA; janice_zhao@urmc.rochester.edu (J.Z.); brendan_guercio@urmc.rochester.edu (B.J.G.)

**Keywords:** prostate cancer, chemotherapy, oncology, docetaxel, cabazitaxel, mitoxantrone, castration-sensitive, castration-resistant

## Abstract

**Simple Summary:**

Prostate cancer is the second most frequently occurring cancer in men, and patients with advanced prostate cancer have poor long-term survival. Chemotherapy is the cornerstone of systemic therapy for advanced prostate cancer and can prolong survival, both in castration-sensitive and castration-resistant disease. In this review, we summarize the data that underlie the integration of chemotherapy into the management of advanced prostate cancer, including data supporting the combination of chemotherapy with next generation androgen receptor signaling inhibitors.

**Abstract:**

Prostate cancer is the second most common cancer among men. Despite advances in diagnosis and management, prostate cancer led to more than 300,000 deaths globally in 2020. Chemotherapy is a cornerstone of therapy for advanced prostate cancer and can prolong survival of patients with both castration-sensitive and castration-resistant disease. Herein, we present a comprehensive review of the data supporting implementation of chemotherapy in the modern treatment of advanced prostate cancer, with special attention to the use of chemotherapy for aggressive variant prostate cancer (e.g., neuroendocrine prostate cancer) and the combination of chemotherapy with androgen signaling inhibitors. As the field of prostate cancer research continues to rapidly evolve yielding novel agents and treatment modalities, chemotherapy continues to play an essential role in prolonging the survival of patients with advanced and metastatic prostate cancer.

## 1. Introduction

Prostate cancer is the second most commonly diagnosed cancer among men and the fourth most commonly diagnosed cancer overall in the world, with 66 years the average age at diagnosis [1,2]. In 2020, there were an estimated 1,414,259 new cases of prostate cancer globally with an age standardized incidence rate (ASIR) of 30.7 per 100,000 males. There were an estimated 375,304 prostate cancer-related deaths with an age standardized mortality rate (ASMR) of 7.7 per 100,000 males [3]. In the United States, there will be an estimated 288,300 new prostate cancer diagnoses and 34,700 deaths from prostate cancer in 2023 [4]. 

Prostate cancer refers to malignancy originating in the prostate gland, most commonly adenocarcinoma, although rarely, other forms such as small cell/neuroendocrine carcinoma can occur. Metastatic prostate cancer is defined by the spread of prostate cancer to locations outside of the prostate. The most common sites of metastatic disease are lymph nodes and the axial skeleton, although more aggressive and advanced disease can metastasize to other sites such as the liver and lungs [5]. While localized prostate cancer can be cured, metastatic prostate cancer is usually incurable, and therefore, the goal of treatment for metastatic prostate cancer is to control disease for as long as possible to prolong life and maintain quality of life. However, given enough time, metastatic prostate cancer will often develop resistance to standard therapies and progress to a terminal state. Uncontrolled metastatic prostate cancer can cause severe complications, including but not limited to bone pain, pathologic fractures, spinal cord compression, and death [5,6,7]. Prostate specific antigen (PSA) is a protein produced specifically by both benign and malignant prostate cells, and measurement of PSA in the blood serves as a reference marker to guide the diagnosis and monitoring of prostate cancer, including assessment of response to treatment [8,9]. Indeed, guidelines have even recommended the measurement of PSA as one means of defining disease progression in clinical trials [10].

Androgen deprivation therapy (ADT) has long been the backbone of therapy for advanced prostate cancer [11]. Prostate cancer, whether localized or metastatic, is uniquely dependent on androgens to fuel its growth [5]. Therefore, all standard systemic treatment regimens for metastatic prostate cancer include androgen deprivation therapy (ADT) as a component, usually in the form of medical castration with gonadotropin-releasing hormone agonists or antagonists that halt androgen production by the testicles to maintain circulating testosterone levels below 50 ng/dL [6,12]. 

Accumulating evidence has demonstrated that select patients with metastatic prostate cancer can benefit from addition of other therapies to ADT. Notably, chemotherapies specifically refer to cytotoxic systemic medications that promote cell death by inhibiting rapidly dividing cells, and are considered a modality of therapy distinct from the hormonal anti-androgen medications that are also used to treat prostate cancer [5,6,7]. In the 1990s, the role of chemotherapy in metastatic prostate cancer emerged. Additional developments in the last decade have further shaped the contemporary treatment of this disease [13,14,15,16]. Currently, chemotherapy is approved by the Food and Drug Administration (FDA) and the European Medicines Agency (EMA) in the treatment of both metastatic castration-sensitive prostate cancer (mCSPC) and metastatic castration-resistant prostate cancer (mCRPC) [7]. 

As will be explored in detail during the course of this review, chemotherapies commonly utilized in the treatment of metastatic prostate cancer include the taxane chemotherapies docetaxel and cabazitaxel, which reversibly bind to microtubulin to inhibit microtubule depolymerization and thereby prevent cell division and promote cell death [7,17]. In select cases, additional androgen-directed therapies, such as the androgen receptor inhibitor darolutamide or the 17α-hydroxylase/C17,20-lyase inhibitor abiraterone, can be added to these taxane chemotherapies [7]. While androgen-directed therapies such as darolutamide and abiraterone are not cytotoxic chemotherapies themselves, the addition of such hormonal therapies to cytotoxic chemotherapy plus ADT can improve treatment efficacy in specific clinical settings [18,19]. Other chemotherapies that can be used in select cases of metastatic prostate cancer include the topoisomerase II inhibitor mitoxantrone and platinum-based chemotherapies such as carboplatin and cisplatin, all of which promote cancer cell death by damaging the DNA of rapidly dividing cells [7,20].

The objective of this review is to discuss the data supporting the current incorporation of chemotherapy for the treatment of prostate cancer. We will focus on clinical trials that have informed current chemotherapy practices for metastatic prostate cancer, often referring to these trials by name. For ease of reference, a list of commonly used titles of clinical trials referred to throughout the text are provided in Table 1. Notably, the acronyms used to name these clinical trials are often designed to create a convenient short-hand, such that the acronyms do not always accurately abbreviate the full title of the trial.

## 2. Metastatic Castration-Resistant Prostate Cancer

### 2.1. Mitoxantrone

In 1996, mitoxantrone, a synthetic anthracenedione that intercalates into DNA and inhibits topoisomerase II, became the first FDA approved chemotherapy for the treatment of mCRPC [13,20]. In a phase III study reported by Tannock et al., 161 patients with symptomatic mCRPC were randomized to receive either mitoxantrone 12 mg/m^2^ every three weeks plus prednisone 5 mg twice daily or prednisone 5 mg twice daily alone [13]. The primary end point for the study was a palliative response with a decrease in pain defined as a two-point reduction in pain on a six-point pain scale without increased analgesic medications. Of the patients who received mitoxantrone plus prednisone, 29% (23/80 patients) (95% confidence interval [CI] 19–40%) experienced a palliative response compared to 12% (10/81 patients) (95% CI 6–22%) of the patients who received prednisone alone (*p* = 0.01). There was no difference in overall survival (OS) (*p* = 0.27). Of the 130 patients who received mitoxantrone, which included crossover, there were nine cases of febrile neutropenia [13]. 

The role of mitoxantrone for symptom palliation was further supported by the Cancer and Leukemia Group B (CALGB) 9182, a phase III trial [21]. Patients with mCRPC were randomly assigned to receive either mitoxantrone 14 mg/m^2^ every three weeks and hydrocortisone 30 mg in the morning and 10 mg in the evening or hydrocortisone alone. The primary endpoint of the study was survival duration, but similar to the results reported by Tannock et al., there was no difference in overall survival with mitoxantrone plus hydrocortisone (12.3 months) compared to hydrocortisone alone (12.6 months, *p* = 0.77) [13,21]. The findings, however, did suggest that patients who received mitoxantrone plus hydrocortisone experienced greater improvement in quality of life, especially in regards to pain control. For example, two items from the symptom distress scale evaluating pain, “how often” and “how severe,” favored the mitoxantrone plus hydrocortisone group (*p* = 0.06 and *p* = 0.03, respectively) [21]. There have been subsequent studies, which will be discussed, that demonstrated the improvement in survival for patients with metastatic prostate cancer treated with chemotherapy [14,16,31]. Mitoxantrone remains an option for symptomatic patients with mCRPC who have previously received docetaxel and may not be able to tolerate other therapies, though the increasing number of therapeutic options for mCRPC has made its use less frequent over time [7]. 

### 2.2. Docetaxel

The survival benefit of docetaxel, a semisynthetic taxane, compared to mitoxantrone was demonstrated by the phase III study TAX 327 [14,31,32]. In this study, 1006 patients with mCRPC were randomized to receive either mitoxantrone 12 mg/m^2^ every three weeks, docetaxel 75 mg/m^2^ every three weeks, or docetaxel 30 mg/m^2^ weekly for five of every six weeks. Patients in all three arms received prednisone 5 mg twice daily. The primary end point was overall survival, and secondary end points were pain, PSA levels, and quality of life. The group receiving docetaxel every three weeks had longer survival with a median survival of 19.2 months (95% CI, 17.5–21.3 months) compared to the mitoxantrone group survival of 16.3 months (95% CI, 14.3–17.9 months) (*p* = 0.004). The group given docetaxel every three weeks, compared to the group given mitoxantrone, also had higher rates of PSA response (45% vs. 32%, *p* < 0.001), more frequent reduction in pain (35% vs. 22%, *p* = 0.01), and more frequent improvement in quality of life (22% vs. 13%, *p* = 0.009). Although weekly docetaxel also had greater improvement in quality of life compared to mitoxantrone, there was no statistically significant difference in survival between weekly docetaxel and mitoxantrone. Compared to those who received mitoxantrone, patients who received docetaxel every three weeks more commonly experienced adverse events of fatigue, alopecia, diarrhea, nail changes, sensory neuropathy, change in taste, stomatitis, and peripheral edema (*p* ≤ 0.0015), as well as increased grade 3 or 4 neutropenia and dyspnea (*p* ≤ 0.05). However, patients receiving docetaxel experienced less impairment in left ventricular ejection fraction (*p* ≤ 0.0015). TAX 327 helped to establish docetaxel 75 mg/m^2^ as first-line chemotherapy in patients with mCRPC, and docetaxel was FDA approved for treatment of mCRPC in 2004 [14,31,33]. 

### 2.3. Cabazitaxel

In 2010, cabazitaxel, a taxane, was approved by the FDA for use in the treatment of patients with mCRPC who previously had been treated with docetaxel based on the TROPIC study, a phase III trial in which 775 men with mCRPC whose disease had progressed during or after treatment with docetaxel were randomized to receive either mitoxantrone 12 mg/m^2^ or cabazitaxel 25 mg/m^2^ (C25) every three weeks along with 10 mg prednisone daily [16,17]. The primary endpoint was overall survival and secondary endpoints were progression-free survival and safety. The median overall survival of the cabazitaxel group was 15.1 months (95% CI 14.1–16.3) versus 12.7 months (95% CI 11.6–13.7) in the mitoxantrone group, with a hazard ratio (HR) of 0.70 (95% CI 0.59–0.83, *p* < 0.0001). Median progression-free survival was 2.8 months (95% CI 2.4–3.0) among those who received cabazitaxel versus 1.4 months (95% CI 1.4–1.7) among those who received mitoxantrone, with an HR of 0.74 (95% CI 0.64–0.86, *p* < 0.0001). Cabazitaxel was also shown to have more favorable tumor response rates (14.4% vs. 4.4%, *p* = 0.0005) and time to tumor progression (8.8 vs. 5.4 months, *p* < 0.0001) [16]. The most common adverse events associated with cabazitaxel were hematological and included neutropenia, leukopenia, and anemia. Febrile neutropenia occurred in 8% of patients treated with cabazitaxel, and neutropenia-related deaths were reported in 2% of patients (*n* = 7) treated with cabazitaxel [16]. As a result, The National Comprehensive Cancer Network (NCCN) guidelines recommend primary prophylaxis with white blood cell growth factor support for patients with high-risk clinical features for febrile neutropenia receiving cabazitaxel, and consideration of growth factor support for all patients receiving a cabazitaxel dose of 25 mg/m^2^ [12].

The phase III PROSELICA study then compared a reduced dose of cabazitaxel 20 mg/m^2^ (C20) with cabazitaxel 25 mg/m^2^ (C25) [15]. In this non-inferiority study, 1200 patients with mCRPC were randomly assigned to receive either C20 or C25 with prednisone 10 mg daily. Overall survival was the primary endpoint. The median overall survival for patients receiving C20 was 13.4 months compared to 14.5 months among patients receiving C25 (HR, 1.024, one-sided 98.89% upper CI 1.184). The endpoint for non-inferiority was met, and the dose of C20 maintained at least 50% of the survival benefit of C25 demonstrated in TROPIC. C20 appeared to be better tolerated from a safety perspective, with a rate of grade 3 or 4 adverse events of 39.7% compared to a rate of 54.5% for CD25, though white blood cell growth factor support may be needed with either dose [7,15]. For medically fit patients who prefer a more aggressive treatment approach, C25 can be considered [7].

Cabazitaxel has also been compared to the androgen-receptor signaling inhibitors (ARSIs) abiraterone and enzalutamide in the treatment of mCRPC. In the phase III CARD study, 255 patients with mCRPC who previously had received docetaxel and either abiraterone or enzalutamide with progression within 12 months while receiving the ARSI were randomly assigned to receive cabazitaxel 25 mg/m^2^ every three weeks plus prednisone 10 mg daily, abiraterone 1000 mg and prednisone 5 mg daily, or enzalutamide 160 mg once daily [22]. Patients in the ARSI arm received abiraterone if they had previously received enzalutamide and vice versa. The primary end point for this study was imaging-based progression-free survival. Overall survival and progression-free survival were the secondary end points. After a median follow-up of 9.2 months, the median imaging-based progression-free survival was 8.0 months in the cabazitaxel group compared to 3.7 months in the ARSI group (HR 0.54, 95% CI 0.40–0.73, *p* < 0.001). Additionally, overall survival was 13.6 months in the cabazitaxel group compared to 11.0 months in the ARSI group (HR 0.64, 95% CI 0.46–0.89, *p* = 0.008). Grade 3 or higher adverse events occurred in 56.3% of the patients in the cabazitaxel group and 52.4% in the ARSI group. Grade 3 or higher adverse events that occurred more frequently in the cabazitaxel group included asthenia or fatigue, diarrhea, peripheral neuropathy, and febrile neutropenia. Meanwhile, grade 3 or higher adverse events that occurred more frequently in the ARSI group were renal disorders, musculoskeletal discomfort or pain, cardiac disorders, and spinal cord or nerve-root disorders [22]. On the basis of the data above, cabazitaxel is an established treatment option for mCRPC in the third line and beyond [7].

Although docetaxel has been established as standard first-line chemotherapy in patients with mCRPC, cabazitaxel can be considered as an alternative first-line therapy for those who may not be candidates for docetaxel [7]. The phase III FIRSTANA trial randomized 1168 patients with chemotherapy-naïve mCRPC to receive cabazitaxel 20 mg/m^2^, cabazitaxel 25 mg/m^2^, or docetaxel 75 mg/m^2^ every three weeks plus prednisone 10 mg daily [26]. The primary end point of the study was overall survival. The estimated median overall survival was 24.5 months, 25.2 months, and 24.3 months in the C20, C25, and docetaxel arms, respectively. Neither of the hazard ratios for cabazitaxel (C20 or C25) versus docetaxel was statistically significant. The rate of grade 3 or 4 adverse events in the C20 group was 41.2% compared with 60.1% for C25, and 46% for docetaxel. Febrile neutropenia, neutropenic infection, hematuria, and diarrhea were more frequent among those receiving C25. Meanwhile, peripheral neuropathy, edema, alopecia, and nail disorders were more frequent among those receiving docetaxel. The rates of treatment discontinuation due to adverse events were 25.2%, 31.7%, and 33.9% with C20, C25, and docetaxel, respectively. Cabazitaxel and docetaxel were found to have different toxicity profiles, with there being numerically fewer grade 3 or 4 adverse events and less adverse events-related treatment discontinuation with C20. There were no statistically significant differences in overall survival, progression-free survival, PSA response, or pain response among the treatment arms, and so cabazitaxel was not considered to be superior to docetaxel [26]. As per NCCN guidelines, cabazitaxel can be considered as an option for first-line chemotherapy in patients with mCRPC who may not be candidates for docetaxel and/or for those who may need to avoid docetaxel’s toxicity profile, such as those with symptomatic peripheral neuropathy [7]. Table 2 includes phase III trials evaluating chemotherapy treatments in mCRPC.

Cabazitaxel has also been compared to Lutetium Lu 177 vipivotide tetraxetan (Lu-177-PSMA-617) for the treatment of mCRPC. Lu-177-PSMA-617 is a radiopharmaceutical that delivers beta radiation to prostate specific membrane antigen (PSMA)-positive cells and is approved for the treatment of PSMA-positive mCRPC previously treated with ARSI and taxane-based chemotherapy based on the results of the phase III VISION trial [34,35]. The phase II TheraP study compared Lu-177-PSMA-617 with cabazitaxel in patients with mCRPC who had PSMA-positive disease on gallium-68-PSMA-11 PET-CT scan and had disease progression after docetaxel [36]. In this phase II trial, 200 patients were randomly assigned to Lu-177-PSMA-617 every six weeks up to six cycles or cabazitaxel 20 mg/m^2^ every three weeks up to ten cycles. The primary end point was PSA response rate. There was a PSA reduction of 50% or more from baseline in 65 of 99 (66%) patients (95% CI 56–75) in the Lu-177-PSMA-617 arm versus 37 of 101 (37%) of patients (95% CI 27–46) in the cabazitaxel arm (*p* < 0.0001). Lu-177-PSMA-617 was found to also delay progression (defined as radiographic or PSA progression) compared to cabazitaxel (HR 0.63, 95% CI 0.46–0.86, *p* = 0.0028). Grade 3–4 adverse events occurred in 33% of patients in the Lu-177-PSMA-617 arm compared to 53% of the patients in the cabazitaxel arm. Grade 3–4 thrombocytopenia was more common in patients treated with Lu-177-PSMA-617 (11% vs. 0%), whereas grade 3–4 neutropenia was more common in patients treated with cabazitaxel (13% vs. 4%) [36]. Lu-177-PSMA-617 currently is a treatment option for patients previously treated with taxane-based chemotherapy and an ARSI who have metastatic castration-resistant disease that is predominantly PSMA-positive or have at least one PSMA-positive lesion without dominant PSMA-negative lesions [7].

### 2.4. Combining Taxanes with Platinum Chemotherapy for mCRPC

The phase II RECARDO trial investigated taxane-platinum combination therapy in mCRPC [29]. In this study, docetaxel plus carboplatin was compared to docetaxel re-treatment in patients who progressed after initial response to docetaxel (with a progression-free interval of at least 3 months after initial docetaxel treatment), and a benefit to combination therapy was not shown. Patients were randomly allocated to receive either docetaxel 75 mg/m^2^ or docetaxel 60 mg/m^2^ with carboplatin AUC 4 every three weeks. All patients received prednisone 5 mg twice daily. Progression-free survival was the primary outcome. The median progression-free survival was 12.7 months (95% CI 9.9–17.5 months) in the docetaxel arm compared to 11.7 months (95% CI 8.5–21.0 months) in the docetaxel plus carboplatin arm (*p* = 0.98). Median overall survival was 18.5 months (95% CI 11.8–24.5 months) in the docetaxel group versus 18.9 months (95% CI 16.0–23.7 months) in the combination therapy group (*p* = 0.79). Grade 3 and 4 infections and gastrointestinal adverse events were significantly higher in the docetaxel plus carboplatin arm versus the docetaxel arm (25% versus 2.7%, *p* = 0.007 and 13.9% versus 0%, *p* = 0.025, respectively) [29].

Cabazitaxel in combination with platinum chemotherapy has also been investigated. Corn et al. conducted a phase I/II study examining the role of cabazitaxel plus carboplatin for the treatment of mCRPC [37]. Patients who had previously received cabazitaxel, carboplatin, or two or more previous chemotherapies were excluded. All patients received growth factor support and oral prednisone 10 mg daily. In the phase I portion of the study, the maximum tolerated doses were cabazitaxel 25 mg/m^2^ and carboplatin AUC 4 mg/mL per min. These doses were chosen for phase II. In the phase II portion, 160 patients with mCRPC were randomly assigned to either cabazitaxel 25 mg/m^2^ or cabazitaxel 25 mg/m^2^ plus carboplatin AUC 4 every three weeks. The primary endpoint was progression-free survival. At a median follow-up of 31.0 months, the median progression-free survival was 4.5 months (95% CI 3.5–5.7) in the cabazitaxel group compared to 7.3 months (95% CI 5.5–8.2) in the cabazitaxel plus carboplatin group with an HR of 0.69 (95% CI 0.5–0.95, *p* = 0.018). The most common grade 3 or higher adverse events included fatigue and cytopenias [37]. 

In this study, patients were stratified for the presence or absence of aggressive variant prostate cancer clinicopathological criteria (AVPC-C). Patients were considered to have met AVPC-C if they had at least one of the seven features: histological evidence of small cell prostate carcinoma, visceral metastases only, predominantly lytic bone metastases, bulky lymphadenopathy or primary tumor with Gleason score of at least 8, low PSA with high volume bone metastases, elevated lactate dehydrogenase (LDH) or carcinoembryonic antigen (CEA), or short interval response to ADT. The subgroup analysis based on stratification demonstrated that the benefit of cabazitaxel plus carboplatin was greater in men with AVPC-C (HR 0.58, 95% CI 0.37–0.89, *p* = 0.013). Additionally, the study examined the impact of combination therapy on patients with and without aggressive variant prostate cancer molecular signature (AVPC-MS)-positive tumors. Patients were considered to have AVPC-MS-positive tumors if molecular profiling showed alterations in at least two of the tumor suppressors, TP53, RB1, and PTEN. Patients with AVPC-MS-positive tumors treated with cabazitaxel had a median progression-free survival of 2.2 months (95% CI 1.7–3.0) compared to 6.0 months (95% CI 4.4–8.2) in patients treated with cabazitaxel and carboplatin (*p* = 0.00033), and a median overall survival of 9.9 months (95% CI 8.5–13.8) compared to 17.4 months (95% CI 11.2–29.5, *p* = 0.0024). However, patients with AVPC-MS-negative tumors did not derive significant benefit from the addition of carboplatin, wherein those treated with cabazitaxel had a progression-free survival of 5.9 months (95% CI 5.1–7.4) compared to 6.0 months (95% CI 4.5–8.1) for those treated with cabazitaxel plus carboplatin (*p* = 0.74) and a median overall survival of 22.2 months (95% CI 20.3–31.0) compared to 18.9 months (95% CI 11.2–27.3), respectively (*p* = 0.19). The results from this study suggest that patients with aggressive variant prostate cancer may experience survival benefit with the addition of carboplatin to cabazitaxel, while those without AVPC may be at risk of increased adverse events without the benefits of extended survival [37]. NCCN clinical guidelines suggest that cabazitaxel plus carboplatin can be considered in healthy patients with aggressive variant prostate cancer. Of note, when using cabazitaxel in combination with carboplatin, the dosing suggested by NCCN guidelines for cabazitaxel is 20 mg/m^2^ [7]. 

While the RECARDO study did not show benefit in taxane-platinum combination therapy, the study conducted by Corn et al. did so with improvement in progression-free survival [29,37]. There are multiple considerations as for why there may be a discrepancy between the findings of these two studies. RECARDO was discontinued early due to insufficient recruitment after only enrolling 75 of the intended 150 patients. The poor recruitment was attributed to other trials offering new treatment options such as ARSIs as well as patients declining docetaxel re-treatment due to lack of experience or previously experienced side effects. RECARDO, however, did report that based on the post hoc conditional power analysis, a significant difference between the two groups would not have been demonstrated even had the study been completed [29]. Additionally, in RECARDO, patients received docetaxel treatment after having previously received docetaxel, while in the Corn et al. study, patients had not previously received cabazitaxel [29,37]. Another consideration is that in RECARDO, patients in the combination therapy arm received docetaxel 60 mg/m^2^, which is a lower dose compared to the standard 75 mg/m^2^. Finally, occult differences between the two trials in other patient and disease characteristics, such as the frequency of TP53, RB1, and PTEN alterations that were not reported in RECARDO, may have played a role [29,37]. 

## 3. Metastatic Castration-Sensitive Prostate Cancer

### 3.1. ADT Plus Docetaxel Doublet Therapy

Although docetaxel was FDA approved for the treatment of metastatic castration-resistant prostate cancer in 2004, it was not until the 2010s that results of prospectively randomized trials evaluating the role of docetaxel in metastatic castration-sensitive prostate cancer (mCSPC) were reported. [23,27,30]. 

GETUG-AFU 15 was a phase III trial, first published in 2013, that investigated the role of docetaxel in combination with ADT in patients with mCSPC [27]. ADT included orchiectomy or luteinizing hormone-release hormone agonists with the optional addition of non-steroidal anti-androgens. Three hundred eighty-five patients were randomized to receive either ADT or doublet therapy with ADT plus docetaxel 75 mg/m^2^ every three weeks. Patients received up to nine cycles of docetaxel. The primary endpoint for the study was overall survival, and secondary endpoints included progression-free survival. Median follow-up for this study was 50 months. Median overall survival was 58.9 months (95% CI 50.8–69.1) in the ADT plus docetaxel arm and 54.2 months (42.2-not reached) in the ADT arm. The hazard ratio was 1.01 (95% CI 0.75–1.36, *p* = 0.955). There were 72 serious adverse events in the ADT plus docetaxel group, with neutropenia as the most common, occurring in 21% of patients. Additional serious adverse events included febrile neutropenia (3%) and liver function test abnormalities (2%). After two neutropenia treatment-related deaths occurred during study accrual, the data monitoring committee recommended granulocyte colony-stimulating factor after docetaxel treatments, which helped reduce the number of grade 3–4 neutropenia adverse events. No severe adverse events were reported in the ADT only arm, but more frequent adverse events in this group included hot flashes, decreased libido, erectile dysfunction, and anemia. The initial report of this study suggested docetaxel with ADT should not be used first-line in the treatment of mCSPC as the addition of docetaxel did not improve overall survival [27]. 

In the phase III CHAARTED trial that was published in 2015, 790 patients with mCSPC were randomly assigned to receive either ADT with docetaxel 75 mg/m^2^ every three weeks for six cycles or ADT alone [23]. Patients were not required to receive daily prednisone. Patients in the study were stratified according to high volume or low volume disease (LVD), where high volume disease (HVD) was defined by the presence of visceral metastases and/or at least four bone lesions with at least one lesion outside the vertebral bodies and pelvis, while LVD was defined by the absence of HVD criteria. The primary endpoint was overall survival. At a median follow-up of 28.9 months, the median overall survival was 57.6 months in the ADT with docetaxel arm versus 44.0 months in the ADT arm. The hazard ratio was 0.61 (95% CI 0.47–0.80, *p* < 0.001). The benefit of ADT with docetaxel was more apparent in HVD patients, with a median overall survival of 49.2 months in the combination group versus 32.2 months in the ADT group (HR 0.60, 95% CI 0.45–0.81, *p* < 0.001). Median survival for the LVD group had not yet been reached at the time of initial analysis. Grade 3 or 4 neutropenia occurred in 12.1% of patients receiving combination therapy, while grade 3 fatigue occurred in 4.1% of patients in this group. Both motor and sensory neuropathy occurred in 0.5% of patients in the combination therapy arm [23]. 

The long-term survival analysis of CHAARTED was published in 2018 [38]. After a median follow-up of 53.7 months, the median overall survival was 57.6 months for the group receiving ADT plus docetaxel compared to 47.2 months for the group receiving ADT alone with an HR of 0.72, 95% CI 0.59–0.89, *p* = 0.0018. Furthermore, in patients with HVD, median overall survival was 16.8 months longer in the combination therapy arm compared to ADT alone (51.2 months vs. 34.4 months, HR 0.63, 95% CI 0.50–0.79, *p* < 0.001). Although there was a survival benefit shown for combination therapy in those with HVD, no benefit was identified in those with LVD. The median overall survival was 63.5 months for patients with LVD who received combination therapy versus not reached for those who received ADT alone (HR 1.04, 95% CI 0.70–1.55, *p* = 0.86). Thus, the findings from CHAARTED demonstrated a survival benefit with the addition of docetaxel to ADT in patients with high volume disease, but no clear survival benefit with early docetaxel in low volume disease [38]. 

GETUG-AFU 15 reported its long-term survival analysis after a median follow-up of 83.9 months [39]. A post hoc subgroup analysis was performed to evaluate the benefits of ADT plus docetaxel versus ADT alone in high volume disease (HVD) and low volume disease (LVD) patients as defined in CHAARTED [23]. GETUG-AFU 15 found that for patients with LVD, the median overall survival was not reached in the ADT plus docetaxel group (95% CI 69.5-NR) compared to 83.4 months (95% CI 61.8-NR) in the ADT group with a hazard ratio of 1.02 (95% CI 0.67–1.55, *p* = 0.9), indicating no significant difference in overall survival among LVD patients. For patients with HVD, the median overall survival was 39.8 months (95% CI, 28.0–53.4) in those receiving ADT plus docetaxel compared to 35.1 months (95% CI, 29.9–43.6) in those receiving just ADT. The hazard ratio for overall survival was 0.78 (95% CI 0.56–1.09, *p* = 0.14), suggesting a 22% reduction in the risk of death in patients with HVD. Although this was not statistically significant, the study was not initially designed for subgroup analysis, and insufficient statistical power may have played a role. Based on this analysis, GETUG-AFU 15 suggested that there may be survival benefit with early docetaxel in patients with mCSPC that have high volume disease [39].

Therefore, GETUG-AFU 15 showed a non-significant reduction in risk of death in patients with HVD who received ADT plus docetaxel, whereas CHAARTED showed a statistically significant survival benefit in patients with HVD who received combination therapy [38,39]. A potential reason for this discrepancy is the lack of statistical power in GETUG-AFU 15. 

Another trial that investigated the role of docetaxel with first-line ADT in patients with mCSPC was the STAMPEDE trial [30]. STAMPEDE included 2962 patients with high-risk, locally advanced, metastatic or recurrent prostate cancer. Patients were considered high-risk if they met at least two of the following criteria: T3/4, Gleason score of ≥8, or PSA ≥ 40 ng/mL. Patients were randomized to receive either standard of care (hormone therapy for at least two years) only, standard of care plus zoledronic acid (4 mg every 3 weeks for six cycles followed by every four weeks until two years), standard of care plus docetaxel (75 mg/m^2^ every three weeks for six cycles with prednisolone 10 mg daily), or standard of care with both zoledronic acid and docetaxel. At a median follow-up of 43 months, median overall survival was 71 months in the standard of care arm, not reached in the standard of care plus zoledronic acid arm (HR 0.94, 95% CI 0.79–1.11, *p* = 0.450), 81 months in the standard of care plus docetaxel arm (HR 0.78, 0.66–0.93; *p* = 0.006), and 76 months for the standard of care plus zoledronic acid and docetaxel arm (HR 0.82, 0.69–0.97; *p* = 0.022). STAMPEDE did not demonstrate that the addition of zoledronic acid contributed to improvement in survival in mCSPC, but did find that docetaxel improved overall survival [30]. 

STAMPEDE also reported long-term outcomes of standard of care versus standard of care plus docetaxel stratified by metastatic burden for patients with mCSPC [40]. The analysis included 1086 patients with metastatic disease who received lifelong ADT versus ADT with six cycles of docetaxel 75 mg/m^2^ every three weeks and prednisolone 5 mg twice daily. The definition of high-metastatic burden was the same as that used in the CHAARTED trial [23,40]. After a median follow-up of 78.2 months, the median overall survival was 43.1 months and 59.1 months in the ADT and ADT plus docetaxel arms, respectively (HR 0.81, 95% CI 0.69–0.95, *p* = 0.009). For the patients with LVD, median overall survival was 76.7 months in the ADT arm versus 93.2 months in the ADT plus docetaxel arm (HR 0.76, 95% CI 0.54–1.07, *p* = 0.107). For patients with HVD, median overall survival was 35.2 months in the ADT arm vs. 39.9 months in the ADT plus docetaxel arm (HR 0.81, 95% CI 0.64–1.02, *p* = 0.064). Based on its findings, STAMPEDE reported that there is a survival benefit with upfront docetaxel in patients with mCSPC and that this benefit does not differ based on metastatic burden (interaction *p* = 0.827) [40].

### 3.2. Doublet Therapy (ADT Plus Docetaxel) in Low Versus High Volume mCSPC

While there was consensus on the benefits of early docetaxel in high volume disease, GETUG-AFU 15, CHAARTED, and STAMPEDE had differing findings on the role of doublet therapy in low volume disease [38,39,40]. Likely contributing factors for the varying findings among these trials include differences in patient characteristics and treatment. For example, GETUG-AFU 15 had a lower median PSA value. CHAARTED and GETUG-AFU 15 also had fewer de novo mCSPC patients compared to STAMPEDE (Table 3) [38,39,40].

Gravis et al. conducted a meta-analysis of patient subgroups from CHAARTED and GETUG-AFU 15 with the primary end point being overall survival [41]. Findings from this meta-analysis showed that the addition of docetaxel to ADT consistently improved overall survival for patients with HVD (pooled average HR 0.68, 95% CI 0.56–0.82, *p* < 0.001); however, early docetaxel did not appear to improve overall survival in LVD (HR 1.03, 95% CI 0.77–1.38, *p* = 0.8) [41]. Similarly, Botrel et al. conducted a systematic review and meta-analysis of GETUG-AFU 15, CHAARTED, and STAMPEDE evaluating ADT plus docetaxel in mCSPC [23,27,30,42]. ADT with docetaxel demonstrated increased overall survival compared with ADT alone (HR = 0.73, 95% CI 0.64–0.84, *p* < 0.0001) with moderate heterogeneity (Chi^2^ = 3.84; degrees of freedom = 2 [*p* = 0.15]; I^2^ = 48%). The benefit in overall survival was more apparent in patients with HVD receiving ADT plus docetaxel (HR = 0.67, 95% CI 0.54–0.83, *p* = 0.0003). The pooled analysis for patients with LVD showed no statistically significant difference in overall survival between patients who received ADT plus docetaxel compared to ADT alone (HR = 0.87, 95% CI 0.61–1.23, *p* = 0.42) [42]. NCCN guidelines currently suggest ADT with docetaxel, as well as either abiraterone or darolutamide, for patients who are fit for chemotherapy and have high volume metastatic disease [7].

### 3.3. ADT Plus Docetaxel and ARSI Therapy

In 2022, the FDA approved the use of darolutamide, an ARSI, in combination with docetaxel for patients with mCSPC [43]. This approval was based on the phase III ARASENS trial, in which 1306 patients with mCSPC were randomized to receive either darolutamide 600 mg twice daily or placebo in combination with ADT and docetaxel [18]. The primary end point for this study was overall survival. At the time of primary analysis, there was a significant improvement in overall survival, and the risk of death was 32.5% lower in the darolutamide triplet therapy arm (HR 0.68, 95% CI 0.57–0.80, *p* < 0.001). Additionally, for the darolutamide arm, the time to development of castration-resistant disease was significantly longer (HR 0.36, 95% CI 0.30–0.42, *p* < 0.001). Grade 3 or 4 adverse events occurred in 66.1% and 63.5% of patients in the darolutamide and placebo groups, respectively. The most common grade 3 or 4 event was neutropenia. ARASENS demonstrated that the addition of darolutamide to ADT and docetaxel increased overall survival with a similar frequency of adverse events compared to placebo with ADT and docetaxel [18].

Darolutamide plus ADT and docetaxel for subgroups by both disease volume and risk was also evaluated in the ARASENS trial [44]. The definitions for HVD and LVD were the same as that of the CHAARTED trial [23]. Meanwhile, disease was considered to be high-risk if there were at least two of the following risk factors: Gleason score ≥ 8, ≥3 bone lesions, or measurable visceral metastases. There was increased overall survival in the darolutamide arm compared to the placebo arm for patients with HVD (HR 0.69, 95% CI 0.57–0.82), as well as for patients with high-risk and low-risk disease (HR 0.71, 95% CI 0.58–0.86 and HR 0.62, 95% CI 0.42–0.90, respectively). Results from ARASENS also suggested a non-significant survival benefit with darolutamide for patients with LVD (HR 0.68, 95% CI, 0.41–1.13). Thus, ARASENS demonstrated that there is overall survival benefit with darolutamide and triplet therapy in patients with HVD as well as both high and low-risk mCSPC [44].

Similarly, the phase III PEACE-1 trial investigated the role of another ARSI, abiraterone, with ADT and docetaxel [19]. In PEACE-1, 1173 patients with de novo mCSPC were randomized to receive standard of care (ADT alone or with docetaxel 75 mg/m^2^ every three weeks), standard of care plus radiotherapy, standard of care plus abiraterone 1000 mg daily and prednisone 5 mg twice daily, or standard of care plus radiotherapy plus abiraterone. Radiographic progression-free survival and overall survival were coprimary endpoints. The median radiographic progression-free survival among patients who received abiraterone in addition to standard of care including docetaxel was 4.46 years compared to 2.03 years among those who received standard of care including docetaxel without abiraterone (adjusted HR 0.50, 99.9% CI 0.34–0.71, *p* < 0.0001). The median overall survival was not reached for the ADT with docetaxel and abiraterone group versus 4.43 years for the ADT with docetaxel group (adjusted HR 0.75, 95.1% CI 0.59–0.95, *p* = 0.017). Grade 3 or higher adverse events occurred in 63% of the patients who received ADT with docetaxel and abiraterone compared to 52% of patients who received ADT with docetaxel. Hypertension and hepatotoxicity occurred more commonly in patients who received abiraterone. Hypertension occurred in 22% of patients who received abiraterone compared to 13% of those who did not receive abiraterone. Hepatotoxicity occurred in 6% of those who received abiraterone with ADT and docetaxel compared to 1% in those who did not receive abiraterone. PEACE-1 showed that triplet therapy with abiraterone improved overall survival and radiographic progression-free survival in patients with de novo mCSPC, with only modest increases in treatment-related toxicity [19].

In the phase III ENZAMET trial, 1125 patients with mCSPC were randomized to receive either the ARSI enzalutamide 160 mg daily or a standard non-steroidal anti-androgen drug in addition to testosterone suppression [25]. Patients were allowed to receive early treatment with docetaxel 75 mg/m^2^ every three weeks for up to six cycles. The primary end point was overall survival. The median follow-up was 34 months at the time of interim analysis. There were 102 deaths in the enzalutamide arm compared to 143 deaths in the standard non-steroidal anti-androgen arm (HR 0.67, 95% CI 0.52–0.86, *p* = 0.002). Results were not affected by adjustments for use of early docetaxel. In the enzalutamide group, 42% of patients experienced a serious adverse event compared to 34% in the standard care group [25].

An updated primary overall survival analysis after a median follow-up of 68 months continued to demonstrate benefit from the addition of enzalutamide. Thirty-seven percent and 48% of patients in the enzalutamide and standard care arms, respectively, had died with a median overall survival that was not reached (HR 0.70, 95% CI 0.58–0.84, *p* < 0.0001) [45]. The overall survival HR when enzalutamide was added to docetaxel and testosterone suppression was 0.73 (95% CI 0.55–0.90) in patients with synchronous metastases, but not metachronous metastases (HR 01.10, 95% CI 0.65–1.86). In the standard care group, 33% had serious grade 3 or 4 adverse events compared to 47% in the enzalutamide group. The most common grade 3 and 4 adverse events were febrile neutropenia associated with docetaxel (6% in the standard care group versus 6% in the enzalutamide group), fatigue (1% vs. 6%) and hypertension (6% vs. 10%). Additionally, seven patients (1%) in the enzalutamide group experienced seizures compared to no patients in the standard care group. The findings of ENZAMET (Table 4) suggested that the addition of enzalutamide should be considered in patients with mCSPC treated with docetaxel [45]. 

### 3.4. ARSI Doublet Therapy Versus Triplet Therapy

While studies have shown benefits of doublet therapy with ADT plus ARSI, as well as benefits of triplet therapy with ADT plus docetaxel and ARSI, doublet therapy with ADT plus ARSI and triplet therapy for mCSPC have not been compared head to head [18,19]. Riaz et al. conducted a living systematic review and meta-analysis of 10 randomized clinical trials consisting of 11,043 patients to evaluate first-line treatment options for mCSPC [46]. The study showed that in the overall population, triplet therapy improved overall survival compared with docetaxel plus ADT; however, triplet therapy was not associated with statistically significant overall survival improvement compared to ARSI doublet therapy. Triplet therapy showed efficacy in the treatment of mCSPC, but also had increased toxicity compared to doublet therapy. Analysis of the study suggested that disease volume and metastatic presentation timing are important considerations when selecting treatment regimen. As an example, patients with HVD may benefit the most from triplet therapy as it may delay disease progression compared with ARSI doublet therapy and docetaxel plus ADT. However, for patients with LVD, there is no evidence that triplet therapy provides a clear benefit compared with ARSI doublet therapy. Patients with LVD appear to have increased treatment benefit with ARSI doublet therapy compared with docetaxel and ADT [46].

## 4. Neuroendocrine Prostate Cancer

Chemotherapy is a cornerstone of therapy for neuroendocrine prostate cancer (NEPC). NEPC is associated with more aggressive clinical course and poorer prognosis. Patients more commonly present with NEPC in the setting of prior hormonal therapies for prostate adenocarcinoma, but patients may also present with de novo disease [47]. Gagnon et al. evaluated prognostic biomarkers and clinical outcomes in NEPC [48]. One hundred thirty-five cases of NEPC were identified, with 59% being treatment-associated. At time of diagnosis, 31% of patients had metastatic castration-resistant disease with a median overall survival of 9.6 months. Anemia and elevated neutrophil to lymphocyte (NLR) ratio > 3 were associated with increased risk of death [48]. Clinical data to guide the management of NEPC are sparse; treatment typically bears a resemblance to the management of small cell lung cancer [49]. Treatment of metastatic NEPC differs from that of prostate adenocarcinoma in that platinum-based chemotherapy regimens are typically used for NEPC. Per NCCN guidelines, treatment suggestions for metastatic NEPC include cisplatin/etoposide, carboplatin/etoposide, docetaxel/carboplatin, and cabazitaxel/carboplatin [7]. In phase 2 studies, response rates for NEPC to platinum plus etoposide-based regimens range from approximately 10 to 60%, with median survival less than one year [50,51,52]. Improving outcomes for patients with NEPC is an area of ongoing research.

A summary of preferred regimens for patients with mCSPC, mCRPC, and neuroendocrine prostate cancer are listed in Table 5. 

## 5. Role of Prednisone in Combination with Docetaxel

Historically, docetaxel for prostate cancer has often been administered with concurrent daily prednisone (or prednisolone), though the importance of concurrent prednisone is not well-established. Prior to the era of modern chemotherapy and ARSIs, prednisone was shown to improve pain relief in patients with metastatic prostate cancer. Tannock et al. found that 38% of patients with prostate cancer and symptomatic bone metastases that had progressed after prior treatment with estrogens and/or orchiectomy reported improvement in pain at one month after starting prednisone [53]. The rationale for use of prednisone in this setting was the potential for glucocorticoid-induced suppression of adrenal androgen production. The study notably found that symptomatic improvement was associated with a decrease in serum concentration of adrenal androgen [53]. Given this data, prednisone was subsequently used in conjunction with mitoxantrone, and later included in trials comparing mitoxantrone to docetaxel [13,14,16,22,26,29,37]. Whether administration of concurrent prednisone with docetaxel is important remains controversial; for example, while STAMPEDE administered prednisolone with docetaxel, CHAARTED did not [23,30]. Notably, corticosteroids have also been shown to inhibit growth of prostate cancer cells through action on various cellular signals, including upregulation of TGF-beta and downregulation of IL-6 [54]. Moreover, in the phase 3 trial of mitoxantrone plus prednisone versus prednisone alone, prednisone 10 mg daily alone resulted in a >50% PSA decline in 24% of patients [55].

Belderbos et al. evaluated the role of prednisone with regards to pharmacokinetics as prednisone is known to be a CYP3A4 inducer, and docetaxel is primarily metabolized in the liver by CYP3A4 and CYP3A5 [56]. The study found that there was no significant difference in docetaxel concentrations among patients who received docetaxel with versus without prednisone. Additionally, the toxicity profiles were similar. Thus, from a pharmacokinetic perspective, docetaxel does not require concomitant administration with prednisone [56].

While prednisone may not affect the pharmacokinetics of docetaxel, it is thought to potentially contribute to docetaxel efficacy. Teply et al. conducted a retrospective study to evaluate the role of prednisone on docetaxel efficacy in patients with mCRPC. In the study, 200 patients were identified who had received either docetaxel with prednisone or docetaxel alone [57]. The cohort who received docetaxel with prednisone had superior progression-free survival (7.8 months versus 6.2 months) with an HR of 0.68 (95% CI 0.48–0.97, *p* = 0.03). Notably, there was no difference in progression-free survival observed among patients pre-treated with abiraterone or ketoconazole, suggesting that benefit was limited to corticosteroid-naïve patients. Thus, prednisone may play a role in augmenting the efficacy of docetaxel in select patients with mCRPC [57]. However, to our knowledge, there are no data demonstrating a benefit from the addition of prednisone to triplet regimens with docetaxel and darolutamide or enzalutamide. Given the potent anti-androgen properties of darolutamide and enzalutamide, it would seem less likely that prednisone would add a meaningful benefit through adrenal suppression. 

## 6. Financial Considerations in the Treatment of Prostate Cancer

The treatment of prostate cancer is not without risks of financial toxicity. Joyce et al. assessed the financial toxicity experienced among patients with metastatic prostate cancer as well as patients’ coping mechanisms for financial toxicity. The study found that 54% of patients with metastatic prostate cancer reported at least some financial hardship. Additionally, a greater proportion of patients who experienced high financial toxicity received infusion therapies compared to those with low financial toxicity (20% vs. 5.3%, *p* < 0.001) [58]. Another study conducted by Joyce et al. found that for patients with advanced prostate cancer with commercial insurance, novel hormonal therapy had significantly higher out-of-pocket costs compared to androgen deprivation monotherapy, with the annual out-of-pocket cost being USD 2581 (95% CI USD 1923–USD 3240) greater with novel hormonal therapy [59]. Thus, financial toxicity is a consideration when determining treatment plan. 

## 7. Future Directions

The role of chemotherapy in prostate cancer is evolving, and there are ongoing clinical trials investigating the role of chemotherapy in the treatment of prostate cancer. A search of the National Institutes of Health ClinicalTrials.gov yielded 10 results when searching for recruiting or not yet recruiting phase III trials for prostate cancer and including the search term “chemotherapy”. On closer review of the search results, there are two phase III trials evaluating the role of chemotherapy treatment in prostate cancer (Table 6) [60]. One ongoing study of particular interest is the DORA trial, which is evaluating docetaxel 75 mg/m^2^ every three weeks for 10 doses with prednisone 5 mg twice daily versus docetaxel 60 mg/m^2^ every three weeks for 10 doses with prednisone along with six injections of radium-223 given at six-week intervals in patients with mCRPC. The primary objective of the study is to compare overall survival [24,61]. 

The role of chemotherapy will undoubtedly evolve as additional therapeutic options are added to the armamentarium of prostate cancer treatments. For example, the role of proteolysis targeting chimeras (PROTAC) in the treatment of prostate cancer is a rapidly developing area of research. PROTACs are heterobifunctional molecules that include a ligand that binds to a protein of interest, a ligand that recruits an E3 ubiquitin ligase, and a linker that connects the two ligands. PROTACs serve as a modality for targeted protein degradation with one potential target being the androgen receptor [62]. Currently, there are three ongoing clinical trials investigating the role of PROTACs to target androgen receptors in the treatment of metastatic castration-resistant prostate cancer: NCT03888612, NCT05067140, NCT04428788 [63,64,65]. How PROTACs and other novel therapeutics will reshape the role of chemotherapy in the management of prostate cancer remains to be seen.

## 8. Conclusions

Over the course of the last three decades, there have been many advances in the treatment of prostate cancer. Chemotherapy, especially docetaxel, has emerged as an important option for patients with metastatic prostate adenocarcinoma. For those with neuroendocrine prostate cancer, which can be particularly aggressive, platinum-based doublet chemotherapy regimens are generally used as first-line treatment [7]. There are ongoing studies investigating the role of chemotherapy in the treatment of prostate cancer, especially in conjunction with other therapies such as ARSIs and radiopharmaceuticals, and there are many exciting opportunities for future research to further advance the treatment of prostate cancer.

## Figures and Tables

**Table 1:** List of abbreviated titles for landmark clinical trials of chemotherapy for metastatic prostate cancer.

Clinical Trial Abbreviation	Full Clinical Trial Title
ARASENS [18]	A randomized, double-blind, placebo-controlled Phase III study of ODM-201 versus placebo in addition to standard androgen deprivation therapy and docetaxel in patients with metastatic hormone-sensitive prostate cancer
CALGB 9182 [21]	Cancer and Leukemia Group-B 9182: hydrocortisone with or without mitoxantrone in men with hormone-refractory prostate cancer
CARD [22]	A phase III, randomized, open label, multicenter study of Cabazitaxel versus an Androgen Receptor (AR)-targeted agent (abiraterone or enzalutamide) in mCRPC patients previously treated with Docetaxel and who rapidly failed a prior AR-targeted agent
CHAARTED [23]	ChemoHormonal Therapy versus Androgen Ablation Randomized Trial for Extensive Disease in Prostate Cancer
DORA [24]	Phase 3 Trial of Docetaxel vs. Docetaxel and Radium-223 for Metastatic Castration-Resistant Prostate Cancer (mCRPC)
ENZAMET [25]	Enzalutamide in First Line Androgen Deprivation Therapy for Metastatic Prostate Cancer
FIRSTANA [26]	Randomized, Open Label, Multi-Center Study comparing Cabazitaxel at 25 mg/m^2^ and at 20 mg/m^2^ in Combination with Prednisone Every 3 Weeks to Docetaxel in Combination with Prednisone in Patients with Metastatic Castration Resistant Prostate Cancer not Pretreated with Chemotherapy
GETUG-AFU 15 [27]	Urogenital Tumor Study Group Association Française d’Urologie 15: Androgen-deprivation therapy alone or with docetaxel in non-castrate metastatic prostate cancer: a randomised, open-label, phase 3 trial
PEACE-1 [19]	Abiraterone plus prednisone added to androgen deprivation therapy and docetaxel in de novo metastatic castration-sensitive prostate cancer: a multicentre, open-label, randomised, phase 3 study with a 2 × 2 factorial design
ProBio [28]	A Biomarker Driven Study in Patients with Metastatic Prostate Cancer
PROSELICA [15]	A Phase III study comparing a reduced dose of cabazitaxel (20 mg/m^2^) and the currently approved dose (25 mg/m^2^) in post-docetaxel patients with metastatic castration-resistant prostate cancer
RECARDO [29]	A randomized phase II trial of docetaxel plus carboplatin versus docetaxel in patients with castration-resistant prostate cancer who have progressed after response to prior docetaxel chemotherapy
STAMPEDE [30]	Systemic Therapy in Advancing or Metastatic Prostate Cancer: Evaluation of Drug Efficacy
TAX 327 [14]	Docetaxel plus prednisone or mitoxantrone plus prednisone for advanced prostate cancer
TROPIC [16]	Prednisone plus cabazitaxel or mitoxantrone for metastatic castration-resistant prostate cancer progressing after docetaxel treatment: a randomised open-label trial

**Table 2:** Phase III trials evaluating chemotherapy treatments in mCRPC.

	Tannock et al. [13] (N = 161)	CALGB 9182 [21] (N = 242)	TAX 327 [14] (N = 1006)	TROPIC [16] (N = 755)	PROSELICA [15] (N = 1200)	CARD [22] (N = 255)	FIRSTANA [26] (N = 1168)
Treatment arms	Mitoxantrone plus prednisone vs. prednisone	Mitoxantrone plus hydrocortisone vs. hydrocortisone	Mitoxantrone vs. docetaxel 75 mg/m^2^ q3 weeks vs. docetaxel 30 mg/m^2^ weekly for 5/6 weeks	Cabazitaxel vs. mitoxantrone	Cabazitaxel 20 mg/m^2^ vs. 25 mg/m^2^	Cabazitaxel vs. ARSI	Cabazitaxel 20 mg/m^2^ vs. 25 mg/m^2^ vs. docetaxel 75 mg/m^2^
Outcome measure	Palliative Response (29% vs. 12%)	Quality of life (improved with mitoxantrone)	OS	OS	OS	OS	OS
HR			0.76 (docetaxel q3 weeks compared to mitoxantrone)	0.70 (cabazitaxel vs. mitoxantrone)	1.024 (C20 vs. C25)	0.64 (cabazitaxel vs. ARSI)	0.97 (C25 vs. docetaxel)
CI			95% 0.62–0.94	95% 0.59–0.83	One-sided 98.89% 1.184	95% 0.46–0.89	95% 0.82–1.16
*p*-value	0.01	0.04	0.009	<0.0001		0.008	0.757
Median follow-up (months)			20.8	12.8		9.2	

ARSI, androgen receptor signaling inhibitor; C20, cabazitaxel 20 mg/m^2^; C25, cabazitaxel 25 mg/m^2^; CI, confidence interval; HR, hazard ratio; OS, overall survival.

**Table 3:** Comparisons among trials of ADT and docetaxel versus ADT alone for patients with mCSPC.

	GETUG-AFU 15 [27,39] (N = 385)	CHAARTED [23,38] (N = 790)	STAMPEDE [30,40] (N = 2962) ^1^
Number of cycles of docetaxel	9	6	6
Gleason score 8 or higher (%)	56.10	61.27	70.63 ^1^
Median PSA level at start of ADT—ng/mL			
Docetaxel + ADT	26.7	50.9	70
ADT	25.8	52.1	67
Hazard ratio for death (ADT + docetaxel vs. ADT)	0.88	0.72	0.81
95% CI	0.68–1.14	0.59–0.89	0.69–0.95
*p*-value	0.3	0.0018	0.009
Median follow-up (months)	83.9	53.7	78.2

^1^ Based on initial STAMPEDE data, which included patients who received ADT plus zoledronic acid and ADT plus zoledronic acid and docetaxel. ADT, androgen deprivation therapy; CI, confidence interval; mCSPC, metastatic castration-sensitive prostate cancer.

**Table 4:** Comparisons among trials evaluating ADT and docetaxel plus ARSI in patients with mCSPC.

	ARASENS [18,44] (N = 1306)	PEACE-1 [19] (N = 710) ^1^	ENZAMET [25,45] (N = 503) ^1^
ARSI	Darolutamide	Abiraterone	Enzalutamide
Number of cycles of docetaxel	6	6	6
Gleason score 8 or higher (%)	78.2	76.90 ^1^	58.31 ^2^
Hazard ratio for death	0.68 (triplet vs. doublet therapy)	0.75 (triplet vs. doublet therapy)	0.82 (enzalutamide vs. standard non-steroidal anti-androgen)
95% CI	0.57–0.80	0.59–0.95	0.63–1.06
*p*-value	<0.001	0.017	--
Median follow-up (months)	43.7	52.8	68 ^2^

^1^ Includes only the population that received or were planned for early docetaxel with ADT (with or without androgen receptor signaling inhibitor). ^2^ In overall trial cohort, including patients with or without planned early docetaxel. ARSI, androgen receptor signaling inhibitor; CI, confidence interval; mCRPC, metastatic castration-resistant prostate cancer.

**Table 5:** Preferred chemotherapy options per NCCN Guidelines ^1^ [7].

mCSPC (for Fit Patients with High-Volume Disease)	mCRPC	Small Cell/Neuroendocrine Prostate Cancer
Docetaxel 75 mg/m^2^ every 3 weeks plus darolutamide, with or without daily prednisone Or Docetaxel 75 mg/m^2^ every 3 weeks plus abiraterone and daily prednisone	For patients with mCRPC who already received docetaxel for mCSPC but have not demonstrated definitive evidence of progression on prior docetaxel in the castration-sensitive setting, docetaxel rechallenge may be considered with: Docetaxel 75 mg/m^2^ every 3 weeks with concurrent steroids ^2^	Cisplatin/etoposide Or Carboplatin/etoposide Or Docetaxel/carboplatin Or Cabazitaxel/carboplatin
	For patients who have not already received docetaxel in the castration-sensitive setting, docetaxel is usually preferred in the castration-resistant setting (prior to cabazitaxel): Docetaxel 75 mg/m^2^ every 3 weeks with concurrent steroids ^2^	
	If cancer progressed despite prior docetaxel, or patients intolerant of or unlikely to tolerate docetaxel: Cabazitaxel 20 or 25 mg/m^2^ every 3 weeks with concurrent steroids ^2^	
	For fit patients with aggressive variant prostate cancer, consider: Cabazitaxel 20 mg/m^2^ plus carboplatin AUC 4 mg/mL/min with concurrent steroids ^2^	

^1^ All regimens are administered in conjunction with androgen deprivation therapy. Addition of growth factor support should be considered for all cabazitaxel containing regimens. ^2^ Concurrent steroids may include dexamethasone on the day of chemotherapy or daily prednisone. AUC, area under the curve; mCSPC, metastatic castration-sensitive prostate cancer; mCRPC, metastatic castration-resistant prostate cancer.

**Table 6:** Ongoing phase III clinical trials involving chemotherapy in prostate cancer treatment ^1^.

ClinicalTrials.gov Identifier	Trial Name	Study Population	Study Objective	Study Arms	Outcome Measures
NCT03574571 [24]	DORA	Patients with mCRPC	Evaluate use of radium-223 along with docetaxel	Docetaxel 75 mg/m^2^ vs. docetaxel 60 mg/m^2^ + radium-223	Primary: Overall survival Secondary: Radiographic progression-free survival Symptomatic Skeletal Event-free survival Time to total ALP progression On-treatment alterations in QOL
NCT03903835 [28]	ProBio	Patients with mCRPC	Evaluate treatments based on biomarker signatures (inferred from diagnostic tissue or liquid biopsy)	Standard of care (with ARSIs, radium-223, cabazitaxel and docetaxel as possible options) vs. enzalutamide, abiraterone, carboplatin, cabazitaxel, docetaxel, or niraparib + abiraterone + prednisone depending on biomarker signature	Primary: Progression-free survival Secondary: Response rate Overall survival Quality of life Cost-effectiveness Safety and tolerability

^1^ Based on ClinicalTrials.gov. ALP, alkaline phosphatase; ARSI, androgen receptor signaling inhibitors; mCRPC, metastatic castration-resistant prostate cancer; QOL, quality of life.
